# Clinicopathological features and differential diagnosis of gastric metastases

**DOI:** 10.1186/s12957-023-03100-y

**Published:** 2023-08-22

**Authors:** Wen Chen, Chengyu Liu, Yuejiao Liu, Jing Yuan, Zhanbo Wang

**Affiliations:** 1 Department of Pathology, The 8th Medical Center, Chinese PLA General Hospital, Beijing, 100091 China; 2 Department of Pathology, The First Medical Center, Chinese PLA General Hospital, Beijing, 100853 China

**Keywords:** Gastric metastases, Clinicopathological features, Differential diagnosis, Immunohistochemistry

## Abstract

**Objective:**

Due to the rarity and non-specificity of symptoms, gastric metastases are often misdiagnosed, and patients are not treated promptly. The aim of this study was to study the clinicopathological features and differential diagnosis of gastric metastases.

**Methods:**

From 2004 to 2021, 14 patients were diagnosed with gastric metastases not resulting from direct invasion (GMNDI) in our hospital, and their imaging and clinicopathological features were analyzed.

**Results:**

PET-CT examination showed hypermetabolic nodules in the stomach. Under gastroscopy, GMNDI showed eminence, nodular or vegetable pattern mass, and ulcer. Microscopically, GMNDI showed similar pathological features and immunophenotypes to the primary tumor. In our study, the most common primary tumors were malignant melanoma (4 cases), small cell lung cancer (3 cases), and hepatocellular carcinoma (3 cases). Immunohistochemistry contributed to the pathological diagnosis and differential diagnosis of gastric metastases. Malignant melanoma expressed HMB45, MelanA, and S-100; small cell lung cancer expressed TTF-1, CD56, and CgA; hepatocellular carcinoma expressed GPC-3, hepatocyte, and Sall4. In a few cases, tumor cells may lose immune markers during metastasis. Therefore, it is necessary to combine medical history, imaging examination, and other clinical diagnosis methods in the pathological diagnosis.

**Conclusion:**

An in-depth understanding of GMNDI is conducive to better diagnosis and treatment planning for gastric metastases and subsequent improvement in patient prognosis.

## Introduction

Gastric cancer is the most common malignancy of the digestive system, accounting for about 5.6% of new cancer cases worldwide [1]. Gastric blood vessels and lymph nodes are abundant, but gastric metastases are very rare, the proportion of which is only 0.2–0.7% [2]. The gastrointestinal symptoms of gastric metastases are nonspecific and mainly include abdominal pain, bloating, and acid reflux, which are similar to the side effects of primary tumor treatment. Imageological examinations are often inconclusive, especially in the early stages of metastasis. Due to the rarity and non-specificity of symptoms, clinicians are more likely to overlook gastric metastases and fail to make a clear diagnosis and timely treatment plan [3]. Especially for gastric metastases of malignant melanoma, early diagnosis, and surgical treatment can significantly improve the prognosis of patients [4]. Therefore, it is of great significance to analyze the characteristics of gastric metastases.

The pathways of tumor involvement in the stomach include peritoneal dissemination, hematogenous dissemination, lymphatic metastasis, and direct invasion [5, 6]. Yang et al. proposed the concept of “Gastric metastases not resulting from direct invasion (GMNDI)” and believed that it had more clinical value [4]. GMNDI refers to the invasion of cancer cells from the primary site into the stomach through blood vessels or lymphatic vessels, excluding direct invasion by tumors of adjacent organs. Common primary tumors include lung, breast, and esophageal cancers. In addition, there are some rare metastases, such as sarcomatoid carcinoma of jejunum, choriocarcinoma, and seminoma [7–9].

Pathology is the gold standard for the diagnosis of GMNDI, and microscopic findings are similar to those of the primary tumor [10, 11]. However, patients’ clinical histories are often vague, and most gastric biopsy specimens are small in size. Most of GMNDI metastases to the gastric submucosa or muscle layer, and the mucosal layer is only slightly involved or not involved [12]. Therefore, the diagnosis of GMNDI can sometimes be difficult in clinical practice. In this paper, we reported 14 cases of GMNDI diagnosed in our hospital and analyzed their clinicopathological features to provide the support for clinical diagnosis and treatment.

## Materials and methods

### Clinical data

From 2004 to 2021, about 15,000 cases of gastric cancer were diagnosed at the First Medical Center and the 8th Medical Center of Chinese PLA General Hospital, of which only 14 cases were GMNDI. All GMNDI cases were diagnosed independently by 3 (associate) chief physicians. The clinical data of the patients were shown in Table 1.

**Table 1:** Clinical features of patients

Patient no	Age (years)	Gender	Primary tumor	Surgery	Other treatment	Time span
1	67	M	Small cell lung cancer	None	Etoposide + atezolizumab	0
2	52	M	Pancreatic adenocarcinoma	Partial gastrectomy	None	24
3	60	M	Cholangiocarcinoma	None	Tislelizumab	0
4	44	M	Malignant melanoma	Partial gastrectomy	None	126
5	54	F	Malignant melanoma	Partial gastrectomy	Temozolomide + cisplatin	96
6	75	M	Malignant melanoma	None	High dose IL-2	0
7	36	M	Malignant melanoma	Partial gastrectomy	Serplulimab	7
8	57	F	Colonic adenocarcinoma	Partial gastrectomy	None	44
9	58	F	Breast cancer	Partial gastrectomy	Docetaxel + capecitabine	72
10	70	M	Hepatocellular carcinoma	None	Intra-arterial chemotherapy	48
11	52	M	Hepatocellular carcinoma	Partial gastrectomy	None	41
12	65	M	Hepatocellular carcinoma	Partial gastrectomy	None	21
13	59	M	Small cell lung cancer	None	Etoposide + cisplatin + serplulimab	0
14	61	M	Small cell lung cancer	None	Etoposide + cisplatin + serplulimab	0

Treatment in the table refers to the treatment of gastric metastases. Time span (month) refers to the time elapsing between primary tumor resection and detection of metastasis

### H&E staining

After fixation with 4% formalin for 12 h, the specimens were sectioned in paraffin. The sections were dewaxed to water and stained with hematoxylin (Sigma) for 3 min. After fully washing with tap water, place them in 1% hydrochloric acid alcohol solution for 30 s. Wash with tap water, return to blue with 0.6% ammonia, and wash with running water. Sections were stained in eosin (Sigma) for 1 min and then dehydrated in gradient. After 100% xylene transparent, the sections were sealed with neutral resin.

### Immunohistochemical staining

Paraffin sections were dewaxed in xylene for 30 min, treated with 3% hydrogen peroxide, and antigen blocked with sheep serum working solution (Zsbio). The primary antibody (Zsbio) was added and incubated at 4 °C for 16 h. After washing with PBS for 3 times, biotin-labeled secondary antibody (Zsbio) was added and incubated at 37 °C for 4 h. After washing with PBS for 3 times, alkaline phosphatase-labeled streptomyces ovalbumin working solution (Zsbio) was added. Wash with PBS for 3 times and develop color for 10 min. Wash gently with tap water and counterstain in hematoxylin solution. After dehydration, transparency, and sealing, the slices were observed under a microscope.

## Results

### Clinical features

**Fig. 1:**
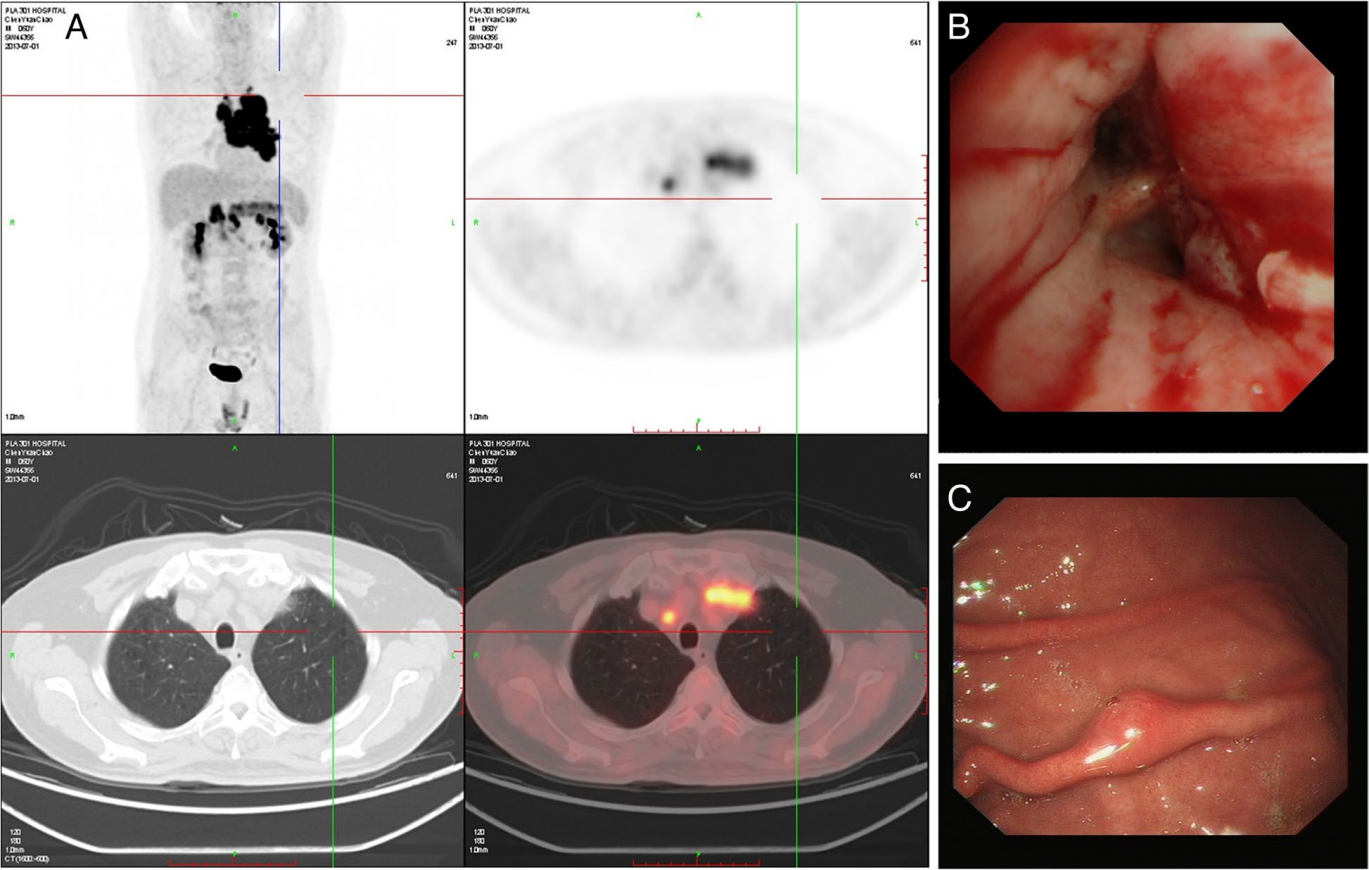
Imaging examination and gastroscopy. **A** PET-CT examination showed hypermetabolic nodules in the stomach and hilus of the lung. **B **Bronchoscopy showed a new organism in the opening of the basal segment of the left inferior lobe. **C** Under gastroscopy, GMNDI showed eminence, nodular or vegetable pattern mass, and ulcer

A total of 14 GMNDI patients were included in this study, with a mean age of 57.85 years, including 9 males and 5 females. Gastric metastases were found synchronously with the primary lesions in 5 patients and metachronously with the primary lesions in 9 patients. The interval time range for the metachronous metastases was 7–126 months. Their clinical symptoms were non-specific, including abdominal pain, distension, and hematochezia. PET-CT examination showed hypermetabolic nodules in the stomach and hilus of the lung (Fig. 1A). Bronchoscopy showed a new organism in the opening of the basal segment of the left inferior lobe (Fig. 1B.). Under gastroscopy, GMNDI showed eminence, nodular or vegetable pattern mass, and ulcer (Fig. 1 C).

### Pathological features

#### Microscopic examination

**Fig. 2:**
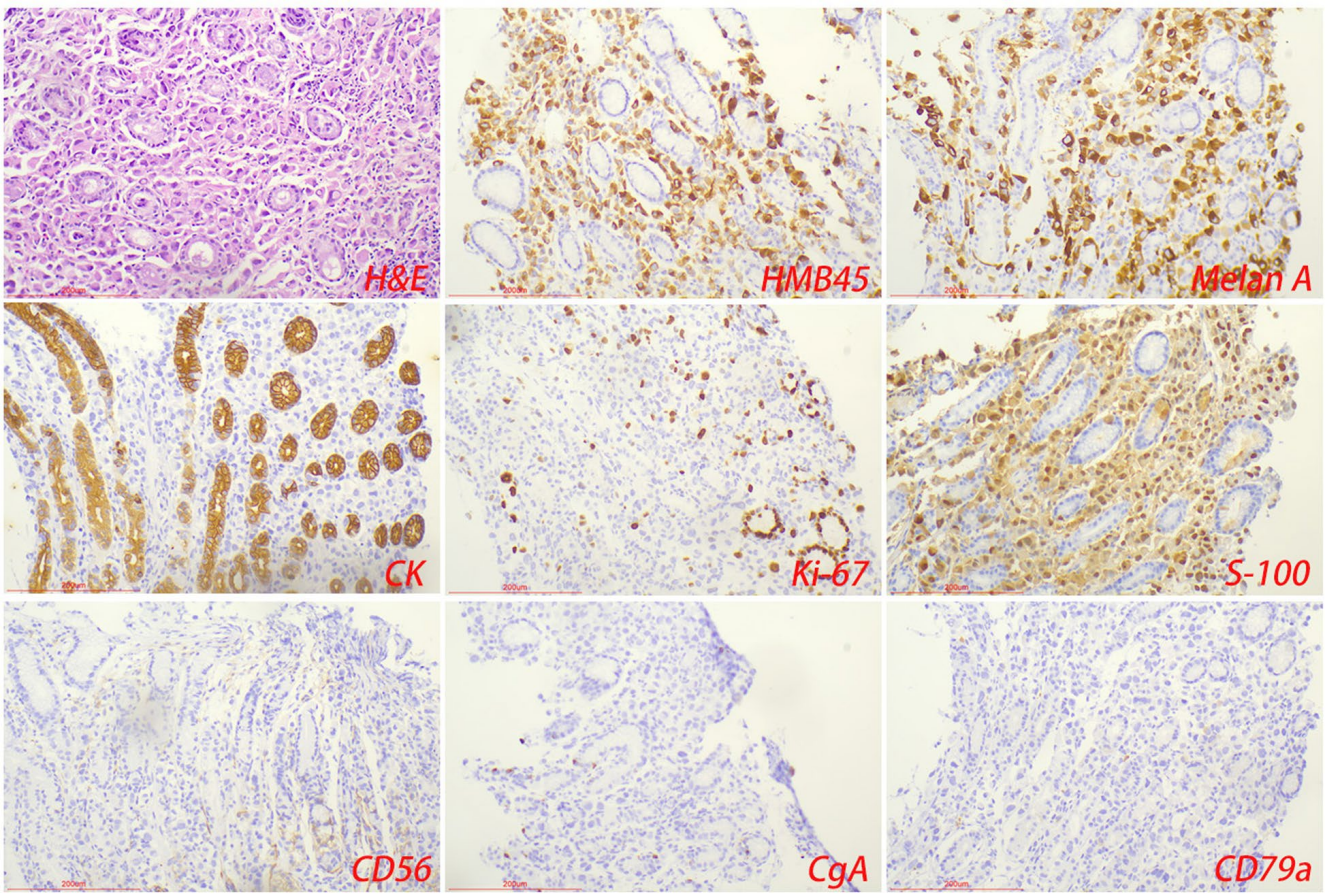
Gastric metastases of malignant melanoma. Tumor cells expressed HMB45, MelanA, and S-100

GMNDI showed similar pathologic features to the primary tumors. In the gastric metastases of hepatocellular carcinoma, the tumor tissue was in the shape of nests, rich in blood sinuses, polygonal tumor cells, basophilic cytoplasm, and visible nucleoli. Among them, the tumors of 2 cases were located in the submucosa, and 1 case was located in the mucosa, which needed to be differentiated from gastric hepatoid adenocarcinoma. In the gastric metastases of colon cancer, hilar cholangiocarcinoma, and pancreatic ductal carcinoma, the tumors were adenoid structures with invasive growth patterns. In the individual case, there appears to be a transition with gastric mucosal glands; it is easy to misdiagnose. In the gastric metastases of breast cancer, the tumor is submucosal, with a nestlike structure and no adenoid area. In this study, 2 cases of gastric metastases of malignant melanoma were infiltrated in the submucosa or muscle layer without mucosal layer infiltration, and 1 case was infiltrated in gastric mucosa with empty cytoplasm, which was difficult to distinguish from signet-ring cell carcinoma (Fig. 2). None of the three cases showed typical pigment particles. In the gastric metastases of small cell lung cancer, a relatively obvious small cell malignant tumor area was found in the gastric biopsy tissue, with small and round cell morphology, local short spindle, little cytoplasm, and some cells showed oat cell-like changes.

#### Immunohistochemical results

**Fig. 3:**
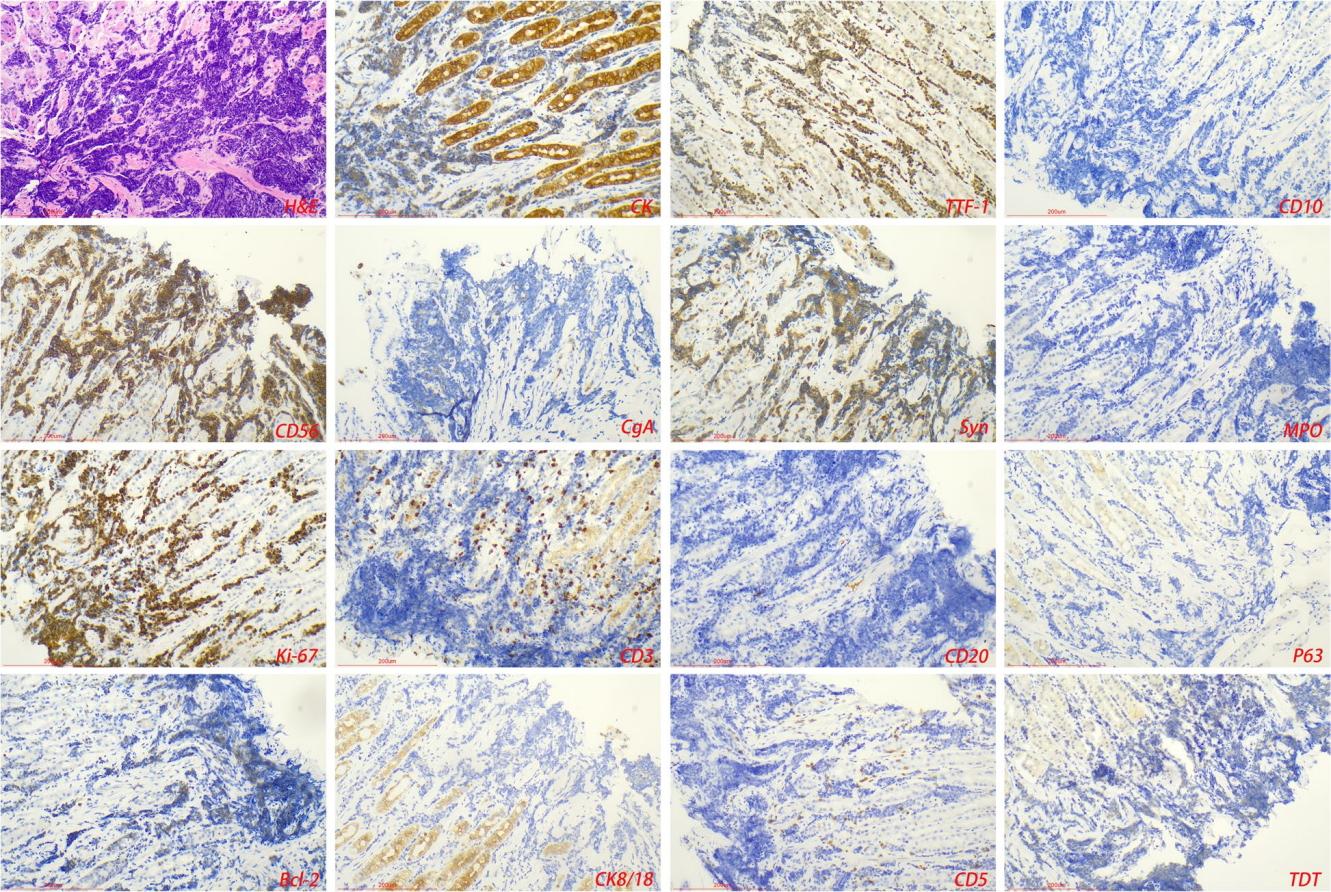
Gastric metastases of small cell lung cancer. Tumor cells expressed neuroendocrine markers (CD56, CgA, or Syn), TTF-1, and CK

**Fig. 4:**
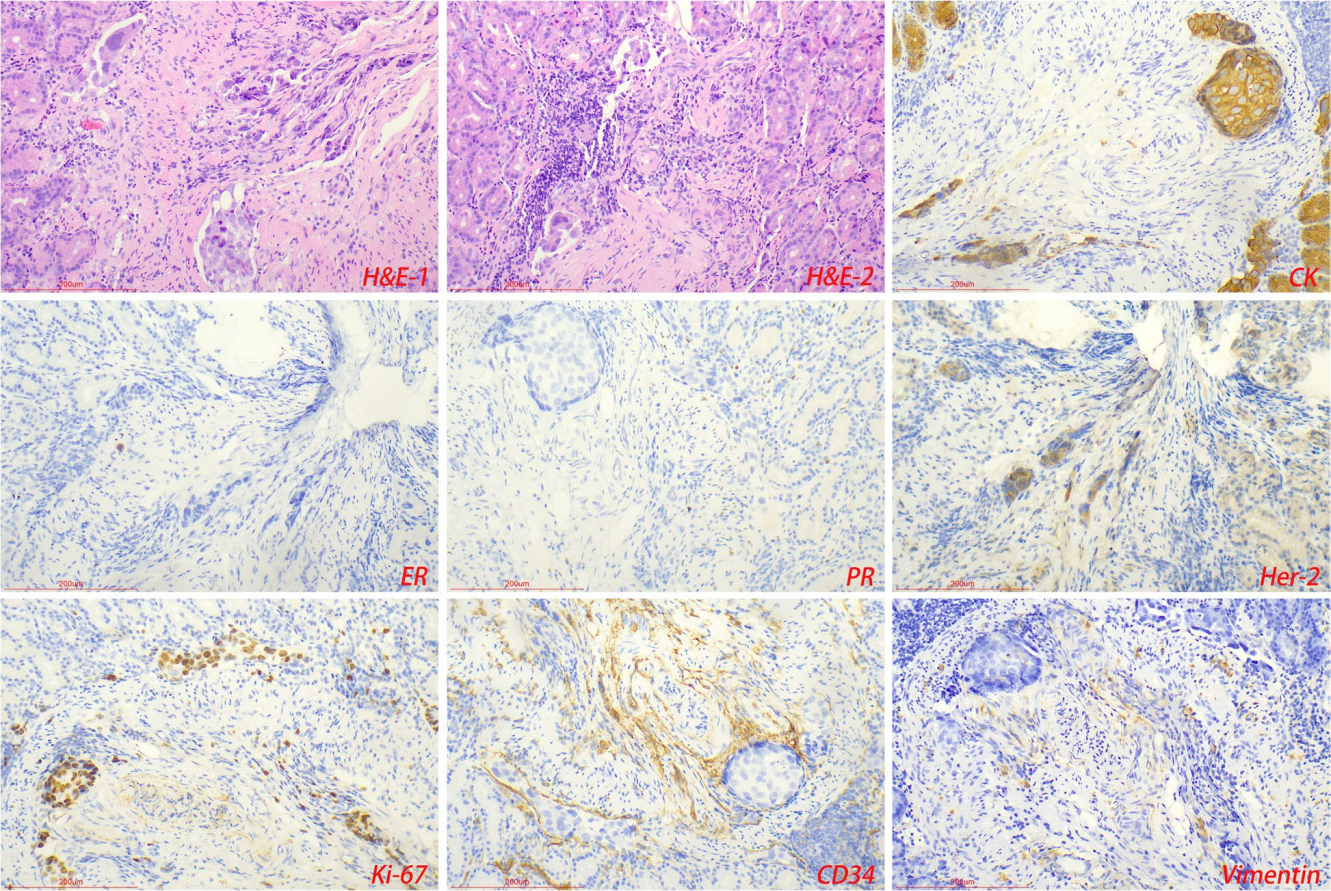
Gastric metastases of breast cancer. Tumor cells expressed Her-2, but ER and PR were not detected

GMNDI often expresses a similar immunophenotype to the primary tumor and can be used to distinguish it from primary gastric tumors. Malignant melanoma expressed HMB45, MelanA, and S-100 (Fig. 2); small cell lung cancer expressed TTF-1, CD56, and CgA (Fig. 3); hepatocellular carcinoma expressed GPC-3, hepatocyte, and Sall4; breast cancer expressed ER, PR, Her-2, and GCDFP-15. CDX2, CK7, CK20, and SATB2 were expressed in colorectal adenocarcinoma. Some GMNDI may lose immune markers during metastasis. For example, in our study, both ER and PR were negative in metastatic lesions of invasive breast cancer (Fig. 4) (Table 2).

**Table 2:** Immunohistochemical antibodies for differential diagnosis of GMNDI

Primary tumor	Antibodies
Small cell lung cancer	CD56, CgA, Syn, CK, TTF-1
Non-small cell lung cancer	NapsinA, TTF-1, P40, CK5, P63
Breast cancer	ER, PR, Her-2, GATA-3, GCDFP-15
Hepatocellular carcinoma	GPC-3, Hepatocyte, Sall4
Malignant melanoma	HMB45, MelanA, S-100
Colorectal adenocarcinoma	CDX2, CK7, CK20, SATB2
Cholangiocarcinoma	CK19, S-100P, MUC-1, HNF-1β
Renal cell carcinoma	CD10, PAX-8, CA9
Ovarian cancer	WT-1, P16
Prostate cancer	PSA, P504S, AR
Germinoma	PLAP, CD30, HCG

## Discussion

Gastric metastases are very rare, the early symptoms are not obvious and not specific, and clinicians are easy to misdiagnose [2]. In our study, half of GMNDIs were diagnosed 1 year after the primary tumor was detected. In some cases, the interval time was longer than 10 years, further complicating diagnosis. GMNDI mostly first metastasizes to the gastric submucosa or muscle layer, rarely directly involving the mucosal layer [13, 14]. However, when obvious clinical symptoms appear, tumor cells have infiltrated the whole layer of the gastric wall, presenting as ulcerated, raised, nodular, or vegetable masses under the gastroscope, which are not significantly different from gastric cancer. Many studies have shown that gastric metastases, mainly from lung, breast, ovarian, and malignant melanoma, metastasize through lymph nodes or blood [15, 16]. In addition, some specific routes of metastasis, such as lung cancer, can be transferred to the stomach by swallowing sputum containing cancer cells. There were some differences between our study and other studies. The most common primary tumors were malignant melanoma, small cell lung cancer, and hepatocellular carcinoma [2].

**Fig. 5:**
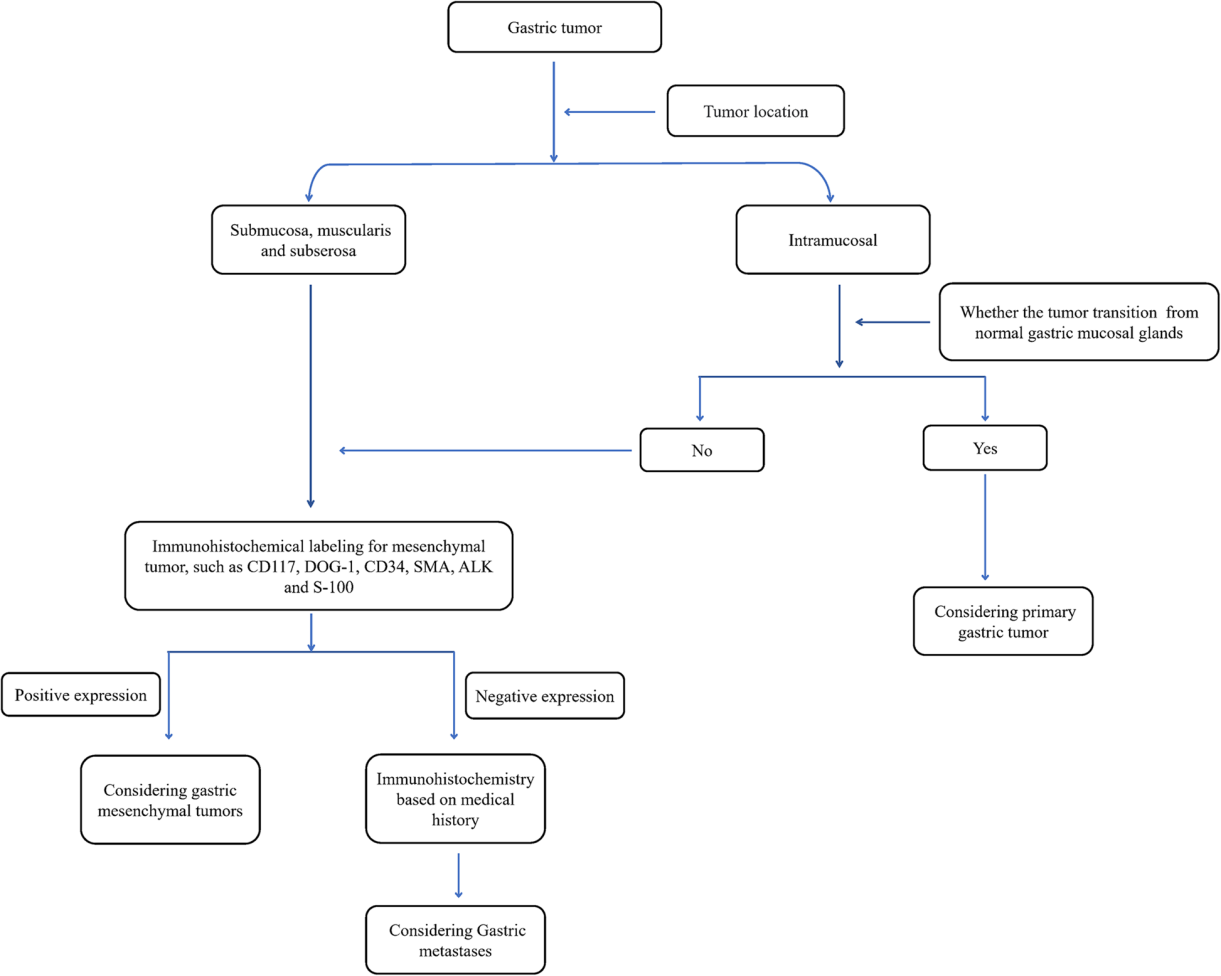
Diagnostic algorithm of pathological diagnosis of gastric metastases

GMNDI often exhibits similar pathological features and immunophenotypes of the primary lesion, which can be used to differentiate from gastric cancer. Microscopically, infiltrative growth of poorly differentiated pigmented cells between gastric glands was observed in the gastric metastases of malignant melanoma. Immunohistochemistry showed the expression of MelanA, S100, and HMB45 in the tumor cells [17, 18]. Gastric metastases of small cell lung cancer show circular, oat, or short fusiform cells with infiltrating growth and obvious interstitial fibrosis. Immunohistochemistry showed that tumor cells expressed neuroendocrine markers (CD56, CgA, or Syn), TTF-1, and CK [19, 20]. In some cases, immunohistochemical assistance is limited, such as gastric metastases of hepatocellular carcinoma and primary hepatoid adenocarcinoma [21]. It should be noted that there may be a loss of immunophenotype during tumor metastasis. In our study, intravascular cancer embolus was observed in gastric metastases of breast cancer, while ER and PR were negative. In addition, immunohistochemical (PD-L1, ALK, or Her-2) and gene detection (EGFR, Kras, and BRAF) in the gastric metastases are conducive to the selection of appropriately targeted drugs and provide an important basis for the formulation of the next treatment plan. In any case, an accurate and detailed medical history is most important for the diagnosis of gastric metastases. Imageological examination and genetic testing are sometimes helpful in assisting diagnosis. Diagnostic algorithm of pathological diagnosis was shown in Fig. 5.

The vast majority of GMNDI occur in the advanced stage of malignancy, so the prognosis for patients is often poor. Clinical studies have shown that the median survival time of gastric metastases of lung cancer patients is only 8 months. Patients’ physical condition, treatment plan, and metastasis of important organs also have a significant influence on the prognosis of patients. At present, surgery, radiotherapy, chemotherapy, and conservative therapy are often used to treat gastric metastases in clinics, and different clinical studies have different results. Y. I. Kim et al. found that total palliative gastrectomy could significantly improve the survival benefits of patients with gastric metastases of lung squamous cell carcinoma [22]. Other studies showed that patients with gastric/duodenal metastases of lung cancer survived longer with conservative treatment compared to surgical treatment [23, 24]. For gastric metastases of breast cancer, systemic therapy is beneficial to the survival of patients, and endoscopic hemostasis can significantly improve the symptoms of gastrointestinal bleeding. When patients appear bleeding, obstruction, or perforation, surgical intervention programs should be taken in time [2, 11].

In conclusion, we reported 14 cases of GMNDI and analyzed their imaging and clinicopathological features. An accurate and detailed medical history is most important for the diagnosis of GMNDI. When the tumor lacks transition to the surrounding normal gastric mucosal glands microscopically, we should consider the possibility of gastric metastasis. Immunohistochemistry can provide an important reference for the diagnosis and differential diagnosis of GMNDI.

## Data Availability

The datasets used and/or analyzed during the current study are available from the corresponding authors on reasonable request.
